# Co-pyrolysis and combustion characteristics of polylactic acid and acrylonitrile-butadiene-styrene: insights into interactions, kinetics and synergistic effects

**DOI:** 10.3389/fchem.2025.1552814

**Published:** 2025-03-31

**Authors:** Xujuan Wu, Yunpeng Yang, Yuanyuan Zhan, Kaiyuan Li, Fei Xiao

**Affiliations:** School of Safety Science and Emergency Management, Wuhan University of Technology, Wuhan, China

**Keywords:** PLA, ABS, co-pyrolysis, thermogravimetric, cone calorimeter, combustion characteristics

## Abstract

Polylactic acid (PLA) and acrylonitrile-butadiene-styrene (ABS) are the most commonly used filaments in 3D printing. To enable filament materials to withstand higher stresses, PLA and ABS are often blended (PLA/ABS). In this work, the co-pyrolysis and combustion properties of PLA/ABS blends of various ratios (75%/25%, 50%/50%, and 25%/75%) were analyzed. Thermogravimetric analysis showed that the catalytic pyrolysis of the blends became more intense as the proportion of PLA in PLA/ABS increased. Cone calorimetry tests indicated that the pyrolysis of ABS determines the peak heat release rate of the PLA/ABS blend. The higher amount of PLA allows the blend to pyrolyze at lower temperatures and the combustion reaction becomes more violent. The theoretical heat of combustion was calculated by correlating the average and maximum HRR with the heat flux through theoretical analysis. The theoretical heat of combustion obtained from the maximum HRR data is more reliable than from the average HRR data. This study has implications for the efficient utilization and fire protection of materials based on PLA/ABS.

## 1 Introduction

To develop a polymer with superior properties for the dynamic market, polymer blending is recognized as a cost-effective and flexible method (Vadori et al., 2017). This approach leverages the complementary properties of various polymers, thereby enhancing the overall performance of blends (Haaparanta et al., 2013). It particularly improves their thermal resistance, flame retardancy, and electrical insulation (Hong et al., 2024). Despite these performance enhancements, research on the thermal stability and combustion properties of polymer blends remains limited, indicating a need for further investigation to fully understand and optimize these polymer blends.

Amidst the dwindling petrochemical resources and escalating environmental pollution (Rochman et al., 2013), biodegradable materials have garnered significant attention in recent years. Polylactic acid (PLA) is a biodegradable and renewable thermoplastic polymer (Scaffaro et al., 2018). PLA exhibits inherent high brittleness, low ultimate elongation, and slow crystallization kinetics, which constrain its widespread application (Al-Itry et al., 2012). The blending of PLA with flexible polymers represents an effective strategy to ameliorate these limitations. Some studies have focused on blends such as PLA/polycarbonate (Lin et al., 2015), PLA/Poly (butylene succinate) (Zhao et al., 2021), and PLA/poly (butylene adipate-co-terephthalate) (Signori et al., 2009). In these studies, both the thermal degradation properties and mechanical properties of these blends were found to be improved compared to the PLA. High-elasticity polymers, such as ABS (Acrylonitrile-Butadiene-Styrene), can substantially improve the fracture toughness of PLA (Dhinesh et al., 2019). When considering tensile strength alone, PLA/ABS (mass ratio of 80%/20%) exhibits superior performance. The incorporation of 20% ABS enhances the blend’s ductility, surpassing the properties of PLA. Flexural test results indicate that an ABS-PLA material with a 50%/50% composition, arranged in alternating layers, demonstrates improved strength compared to single-layer configurations. Research on PLA/ABS primarily concentrates on mechanical properties, while the pyrolytic and combustion behaviors have been less investigated. The low limiting oxygen index (LOI) values for both PLA and ABS polymers suggest a high fire hazard (Jia et al., 2020; Liu et al., 2011), indicating that PLA/ABS blends are highly susceptible to ignition and pose a significant fire safety concern under specific environmental conditions. Therefore, the study of the pyrolytic and combustion behaviors of PLA/ABS could offer valuable data support and guidance for fire safety strategies related to the blend. In a previous study (Wu et al., 2022), the co-pyrolysis kinetics and combustion behavior of PLA/ABS (mass ratio of 50%/50%) were initially investigated. The results showed that PLA promoted the depolymerizations of ABS, thereby enhancing the flammability of the blend. Thus, the study on the pyrolytic and combustion behaviors of PLA/ABS with different ratios is of prospective interest.

This study presents an investigation into the thermal decomposition behavior of PLA/ABS blends with varying ratios at different heating rates, utilizing thermogravimetric analysis. (TGA) The study aims to explore the influence of the PLA to ABS ratio on the pyrolysis characteristics of the blend. Furthermore, the combustion characteristics of PLA/ABS blends with different ratios are analyzed under various radiation heat fluxes (20, 30, and 40 kW/m^2^) employing a cone calorimeter. Thermal decomposition characteristics of PLA/ABS blends were determined using a cone calorimeter, yielding key parameters including ignition time (TTI), mass loss rate (MLR), heat release rate (HRR), and effective heat of combustion (EHC). Correlation analyses were conducted between heat flux density and these characteristic parameters.

## 2 Materials and characterization

### 2.1 Materials and sample preparation

PLA (4032D) and ABS (8391) were manufactured by Nature Works Co., Ltd., United States and Shanghai Gaoqiao Petrochemical Co., Ltd. The PLA/ABS sample preparation formulations are shown in Table 1. The molten mixture of PLA and ABS was blended using a QE-70A type compacting machine from Wuhan Qien Technology Development Co., Ltd. The mixing process was carried out for 4 min at 200°C and 60 rpm. The molten mixture was taken out and then hot-pressed into a standard sample of 100 mm × 100 mm × 3 mm (thickness) at 10 MPa and 180°C for 4 min using a Yangzhou Yuanfeng Experimental Machinery Factory YF-8017 plate vulcanizing machine. For comparison, pure PLA and ABS samples were prepared in the same way.

**TABLE 1:** Compositions of PLA/ABS samples.

Sample name	Composition (wt%)	
	PLA	ABS
P1A0	100	0
P3A1	75	25
P1A1	50	50
P1A3	25	75
P0A1	0	100

### 2.2 Characterization

#### 2.2.1 Thermogravimetric analysis

The thermal decomposition behaviors of the five samples at different heating rates were investigated using the STA6000 simultaneous thermal analyzer from PerkinElmer, United States. The PLA and ABS samples were ground into powder and then weighed approximately 10 mg of powder, placed in an Al_2_O_3_ crucible for the TGA experiment. The experiments were carried out in a high-purity nitrogen atmosphere. The TGA curves were obtained for each sample at heating rates of 5, 10, and 15°C/min, from room temperature to 600°C. The TG experiments were reproducible with an uncertainty of less than 5% (Li et al., 2013).

#### 2.2.2 Cone calorimetry

The combustion behaviors of the five samples were recorded using a cone calorimeter from Motis Fire Technology Co., Ltd. Prior to the cone calorimetry tests, all samples were conditioned in a controlled environment at room temperature (25°C) and a relative humidity of 50% for at least 48 h to ensure consistent initial moisture content. This conditioning process helps to minimize variability in the test results due to differences in sample moisture levels. The 100 mm × 100 mm × 3 mm sample was wrapped in aluminum foil and placed in a holder with insulating wool on the back according to the ISO 5660-1 test standard. The sample was then placed horizontally at a distance of 25 mm from the cone heater. The radiation heat fluxes were 20, 30, and 40 kW/m^2^, respectively. A radiation heat flux of 30 kW/m^2^ represents the growth phase of a real fire, while a heat flux of 20–40 kW/m^2^ is typical in a building fire (Chen et al., 2015b). The critical heat flux, ignition temperature, heat of vaporization, and heat of combustion significantly influence the fire hazard and thermal decomposition processes (Chen et al., 2015a). The cone calorimeter was calibrated before each experiment which was repeated three times to ensure reproducibility (Schartel et al., 2005).

## 3 Results and discussions

### 3.1 Co-pyrolysis behaviors

**FIGURE 1:**
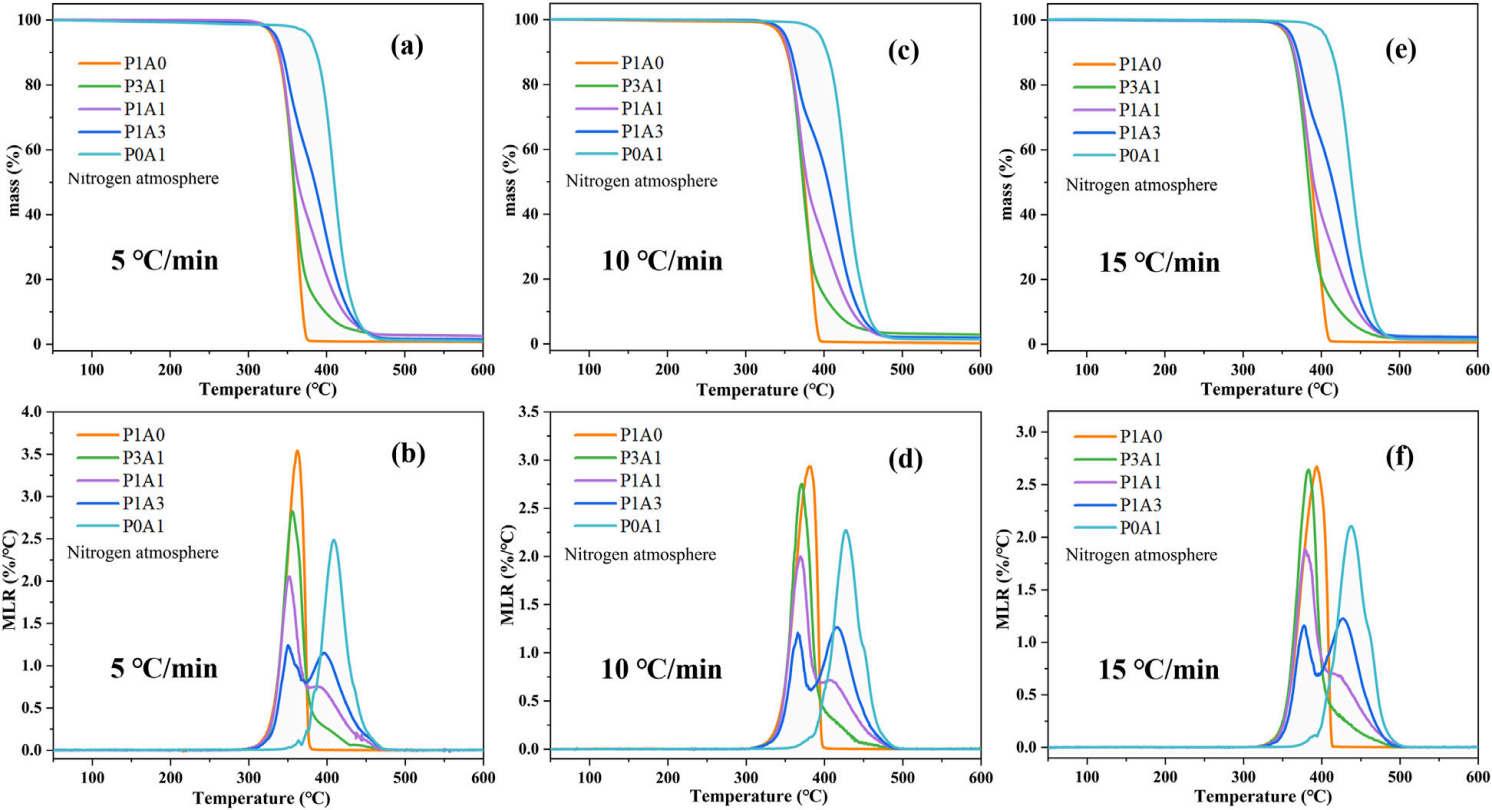
TG curves of **(a)** 5°C/min, **(c)** 10°C/min and **(e)** 15°C/min, and DTG curves of **(b)** 5°C/min, **(d)** 10°C/min and **(f)** 15°C/min for the five samples.

Figure 1 shows the TG and derivative thermogravimetry (DTG) plots for the five samples. From Figure 1, there is only one mass loss stage during the pyrolysis of pure PLA and ABS, which is consistent with the literature (Mandal et al., 2018; Balart et al., 2019). Three PLA/ABS blends exhibit two main stages of mass loss, and the DTG curve has two peaks corresponding to the pyrolysis stages of PLA and ABS. However, ABS makes up a relatively small proportion of P3A1 and its main degradation process is overlap with PLA, resulting in an insignificant second peak. As these two phases of PLA/ABS are derived from PLA and ABS, the TGA data of pure PLA and ABS are compared to the PLA/ABS and are listed in Table 2. In Table 2, the initial temperature of pyrolysis with a total mass loss of 1 wt%, the temperature at maximum MLR in the first and second stages and the completion temperature of pyrolysis when the MLR is close to 0 are defined as *T*_*i*_, *T*_*m1*_, *T*_*m2*_ and *T*_*f*_, respectively. The residues at 600°C are also listed in Table 2.

**TABLE 2:** TGA data for PLA, ABS and PLA/ABS at different mass ratios.

Heat rate (°C/min)	Polymer	T_i_ (°C)	T_m1_ (°C)	T_m2_ (°C)	T_f_ (°C)	Residue at 600 °C (wt%)
5	P1A0	310	362	—	378	0.66
	P3A1	314	355	—	478	2.52
	P1A1	315	352	390	479	2.53
	P1A3	316	349	396	499	1.55
	P0A1	360	—	409	502	1.03
10	P1A0	316	381	—	396	0.19
	P3A1	319	371	—	495	2.83
	P1A1	319	369	407	497	1.73
	P1A3	322	366	416	507	1.88
	P0A1	365	—	427	511	1.39
15	P1A0	330	393	—	412	0.52
	P3A1	330	383	—	497	1.75
	P1A1	330	378	422	508	1.71
	P1A3	330	378	427	517	2.22
	P0A1	374	—	437	519	1.34

For heating rates of 5°C/min, the *T*_*i*_, *T*_*m1*_ and *T*_*f*_ for P1A0 were 310, 362°C and 378°C, respectively, with a residue of 0.66 wt% at 600°C, as shown in Figure 1 and Table 2. For P3A1, the *T*_*i*_, *T*_*m1*_ and *T*_*f*_ were 314, 355°C and 478°C, respectively, resulting in a residue of 2.52 wt% at 600°C. For P1A1, it has a *T*_*i*_ of 315°C, the first and second peaks of DTG appear at 352°C and 390°C respectively, and *T*_*f*_ at 479°C, with a residue of 2.53 wt% at 600C. For P1A3, which has a *T*_*i*_ of 316°C, the first and second peaks of DTG appear at 349°C and 396°C respectively, and *T*_*f*_ at 499°C, with a residue of 1.55 wt% at 600°C. P0A1 has *T*_*i*_, *T*_*m2*_ and *T*_*f*_ of 360, 409°C and 502°C respectively, with a residue of 1.03 wt% at 600°C.

From the data in Figure 1; Table 2, the *T*_*i*_ of PLA/ABS is similar to that of PLA. With the addition of ABS, the second maximum MLR and the *T*_*m2*_ of the mixture tend to increase. This is primarily attributed to the higher thermal stability of ABS. The variation is more significant in that the residue at 600°C is higher for PLA/ABS samples than for PLA and ABS. With an increasing percentage of PLA content, the residue increased. The residue amounts of samples P3A1, P1A1 and P1A3 at 600°C were 1.49 wt%, 1.50 wt% and 0.52 wt% higher than those of ABS, respectively. This suggests that the presence of PLA in PLA/ABS may have catalyzed the reaction and promoted the formation of char.

**FIGURE 2:**
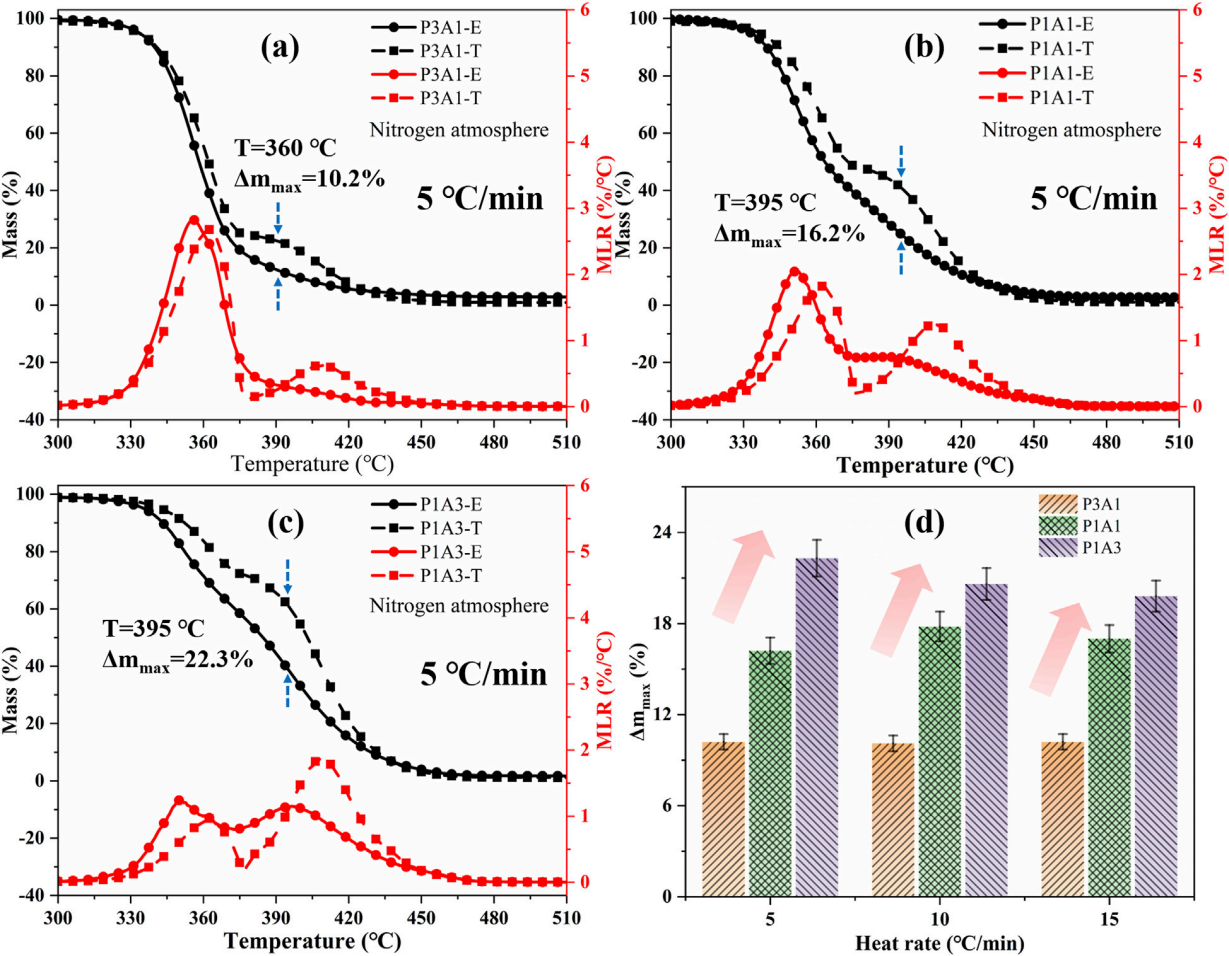
A comparison of theoretical (T) and experimental (E) TG and DTG curves for PLA/ABS blends: **(a)** P3A1, **(b)** P1A1, **(c)** P1A3 and **(d)** Δm_max_.

If there is no interaction between the two plastics, the theoretical mass (WT) is obtained from the mass of pyrolysis alone and the mass ratio of the component in the blend (Kai et al., 2017). It is calculated as:

$$W_{T}=X_{1}W_{1}+X_{2}W_{2}$$ (1)

where *W*_*1*_ and *W*_*2*_ represent the mass loss of PLA and ABS respectively at a given time under the same pyrolysis conditions. *X*_*1*_ and *X*_*2*_ represent the corresponding mass ratios of PLA and ABS. The dashed line in Figure 2 shows the theoretical curve calculated using Equation 1. The difference between experimental and theoretical curves for different heating rates is shown in Figure 2D for the blends. The relevant TGA data for the experimental and theoretical curves are summarized in Table 3. The theoretical and experimental TGA curves do not exactly overlap, while the experimental mass decreases more rapidly. As can be seen from Figure 2; Table 3, the maximum differences in the TGA curves for P3A1, P1A1 and P1A3 were within 10.2%–22.3%, 10.1%–20.6% and 10.2%–19.8%, respectively, at different heating rates. This indicates a large difference between co-pyrolysis and the sum of individual pyrolysis steps.

**TABLE 3:** A comparison of theoretical and experimental TGA data.

Heat rate (°C/min)	Polymer	T_max_ (°C)	Δm_max_ (%)	T_E1_ (°C)	T_T1_ (°C)	T_E2_ (°C)	T_T2_ (°C)
5	P3A1	360	10.2	356	362	—	408
	P1A1	395	16.3	352	363	389	408
	P1A3	395	22.3	350	363	396	408
10	P3A1	379	10.1	370	381	—	427
	P1A1	378	17.8	369	382	407	427
	P1A3	417	20.6	366	383	416	427
15	P3A1	391	10.2	383	393	—	437
	P1A1	390	17.0	378	396	420	437
	P1A3	426	19.8	377	402	427	437

Comparing the peak temperatures in the DTG curves, it can be inferred that the thermal stability of the blends is reduced to some degrees compared to the raw samples. The higher the amount of ABS greater the difference between theoretical and experiment at mass loss curves, and the more obvious the mutual synergistic between PLA and ABS. Theoretically, the DTG of different proportions of PLA/ABS blends will have two peaks that vary proportionally, according to Equation 1. However, as can be seen in Figure 2, the amplitude and temperature of the experimental DTG peaks for ABS in the blends are lower than the theoretical values. The higher the PLA content, the less pronounced the DTG peak of ABS. This indicates that the thermal degradation reaction of ABS in the blend occurs at lower temperatures compared to pure ABS. This suggests that the presence of PLA promotes the pyrolysis of ABS, thereby advancing the onset of its thermal degradation. The difference in PLA and ABS content leads to a variation in the temperature range over which decomposition occurs. The interaction between PLA and ABS results in a synergistic effect on pyrolysis, which is more pronounced than the theoretical results. Specifically, the peak temperature corresponding to PLA pyrolysis decreases more significantly as the ABS content increases. This suggests that ABS not only lowers the maximum MLR temperature of PLA but also enhances the overall pyrolysis process, leading to a more vigorous reaction. These findings provide novel insights into the synergistic interactions between PLA and ABS, highlighting the importance of considering component interactions in polymer blends.

### 3.2 Combustion characteristics

Pyrolysis of materials represents the initial stage of combustion, and the combustion characteristics of the material are crucial for fire modeling and fire safety design (Chen et al., 2015b; Wu et al., 2022). The presence of PLA catalyzed the pyrolysis of ABS, as evidenced by TGA experiments. Therefore, combustion experiments were carried out using a cone calorimeter. Table 4 lists the values of the relevant parameters of TTI, peak of MLR (p-MLR), average of MLR (a-MLR), peak heat release rate (PHRR), maximum average rate of heat emission (MARHE), total heat release (THR) and EHC for the five samples. Correlation analyses were conducted between heat flux density and these characteristic parameters in turn as follows.

**TABLE 4:** Cone calorimetry data.

Heat flux (kW/m^2^)	Polymer	TTI (s)	T_PHRR_ (s)	p-MLR (gs^−1^ m^−2^)	a-MLR (gs^−1^ m^−2^)	PHRR (kW/m^2^)	THR (MJ/m^2^)	EHC (MJ/kg)	MARHE (kW/m^2^)
20	P1A0	74	200	18.6	9.7	326	58	19	168
	P3A1	70	182	29.9	13.8	608	65	21	250
	P1A1	66	176	31.7	13.5	738	72	24	288
	P1A3	65	170	28.8	13.5	758	82	28	321
	P0A1	64	191	25.0	12.4	732	88	32	332
30	P1A0	37	173	24.3	12.1	405	56	18	217
	P3A1	35	132	37.2	14.6	733	62	20	305
	P1A1	33	127	38.5	15.7	876	70	23	344
	P1A3	32	133	33.0	13.3	849	76	27	395
	P0A1	31	137	32.4	12.5	888	84	29	396
40	P1A0	21	119	33.3	15.2	564	56	17	292
	P3A1	20	106	49.0	20.7	1,011	61	19	397
	P1A1	19	102	48.1	18.7	1,053	67	22	440
	P1A3	18	103	42.2	16.9	1,056	72	24	465
	P0A1	17	108	39.0	15.6	1,027	81	27	486

#### 3.2.1 TTI

**FIGURE 3:**
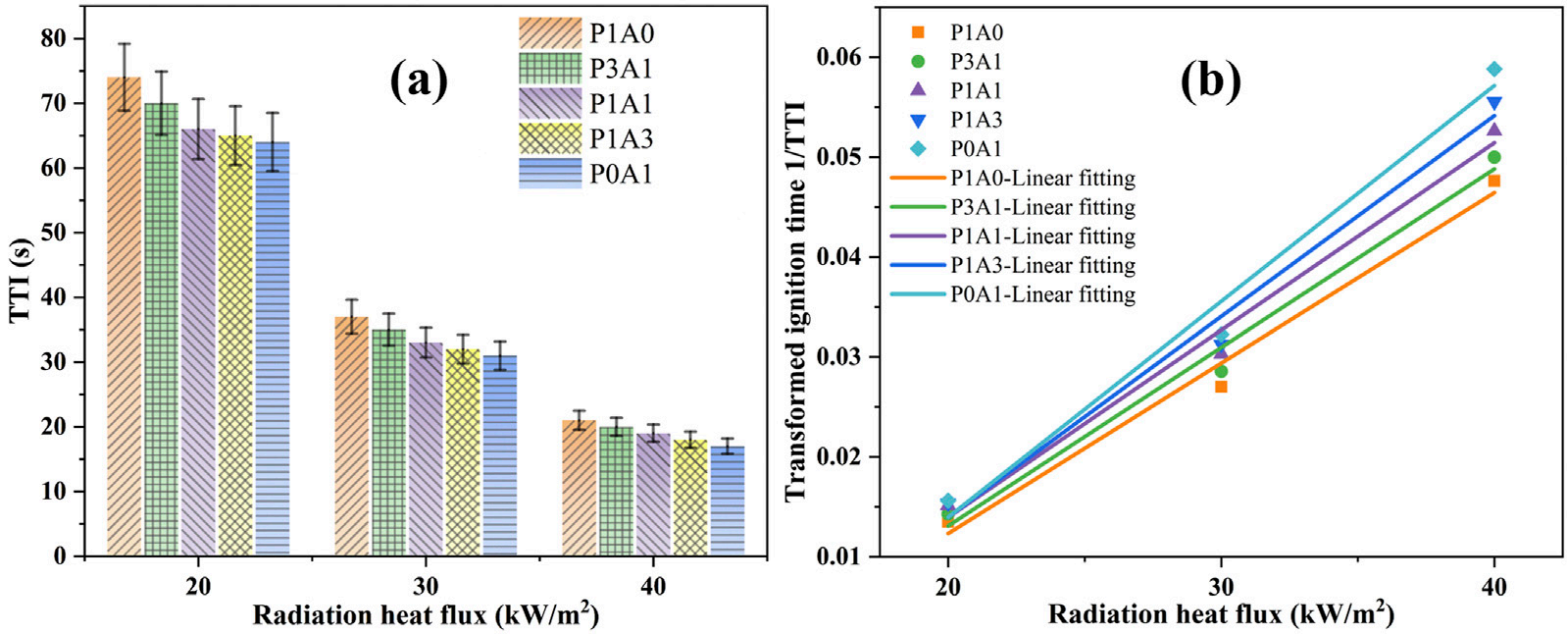
Plot of TTI and 1/TTI versus radiation heat flux: **(a)** TTI and **(b)** 1/TTI.

TTI indicates the degree of fire hazard and flammability of solid combustibles (Quintiere, 2006; Luche et al., 2011; Luche et al., 2012; Quang Dao et al., 2013). Figure 3 shows a diagram of TTI versus radiation heat flux for the five samples. As shown in Figure 3a; Table 4, the TTI decreases with increasing radiation heat flux. With the same radiation heat flux, the TTI of blends with different ratios of PLA and ABS decreases as the percentage of ABS increases. This is mainly attributed to the fact that PLA is more difficult to ignite.

According to Janssens, (1991), the reciprical (1/TTI)^n^ for TTI is firstly related to the heat flux using different values of *n*. *n* is the coefficient, *n* = 0.55 and one corresponding to thermally thick and thermally thin solids, respectively. In the present work, none of the sample sizes were thicker than 3 mm. The samples were identified as thermally thin solids and therefore *n* = 1. The correlation coefficient *R* ^*2*^ was obtained using the least squares method in the correlation equation. The lines fitted to the reciprical TTI versus heat flux for *n* = 1 are shown in Figure 3b. The correlation between conversion TTI and heat flux is shown in Table 5. The *q*_*e*_ *''* indicates the radiation heat flux (kW/m^2^).

**TABLE 5:** TTI-related parameters for five samples.

Polymer	Equation	R^2^	q_cr_'' (kW/m^2^)	q_min_'' (kW/m^2^)	T_ig_ (K)
P1A0	1/TTI = 0.00171 *q*_*e*_ *''* -0.02177	0.99	12.7	18.1	688
P3A1	1/TTI = 0.00179 *q*_*e*_ *''* -0.02262	0.99	12.6	18.0	687
P1A1	1/TTI = 0.00187 *q*_*e*_ *''*- 0.02352	0.99	12.6	18.0	687
P1A3	1/TTI = 0.00201 *q*_*e*_ *''* -0.02619	0.99	13.0	18.5	692
P0A1	1/TTI = 0.00216 *q*_*e*_ *''* -0.02923	0.98	13.5	19.3	701

According to the theory of Quintiere et al. and Luche et al. (Quintiere, 2006; Luche et al., 2011; Luche et al., 2012), the value of critical heat flux (CHF) can theoretically be calculated by

$$CHF=\left[-hboxIntercept/Slope\right]$$ (2)

The value of the intercept of the fitted line with the X-axis (*hboxIntercept*) in Figure 3b is generally considered to be the theoretical critical heat flux (*q*_*cr*_ *''*) using Equation 2. Then the values of *q*_*cr*_ *''* for the P1A0, P3A1, P1A1, P1A3 and P0A1 are 12.7, 12.6, 12.6, 13.0 and 13.5 kW/m^2^, respectively. Delichatsios et al. (Delichatsios et al., 2003; Chen et al., 2016) proposed that the minimum heat flux (*q*_*min*_ *''*) and *q*_*cr*_ *''* conform to Equation 3.

$$q_{cr}^{\prime \prime }=0.7q_{\min}^{\prime \prime }$$ (3)

Then, the *q*_*min*_ *”* can theoretically be calculated as 18.1, 18, 18, 18.5 and 19.3 kW/m^2^, respectively. The ignition temperature of samples can be obtained using Equation 4 from the literature (Chen et al., 2019).

$$\varepsilon q_{\min}^{\prime \prime }=h_{c}\left(T_{ig}-T_{\infty }\right)+\varepsilon \sigma \left(T_{ig}^{4}-T_{\infty }^{4}\right)$$ (4)

where $h_{c}$ is the convective heat transfer coefficient, which is taken as 0.0135 kW m^−2^ K^−1^ in this study (Chen et al., 2015a); ε denotes the surface emissivity of the sample on the ignition, which is taken as 0.88 in this study (Chen et al., 2015a); σ is the Stefan-Boltzmann constant (5.67 × 10^−11^ kWm^−2^ K^−4^); *T*_*ig*_ and $T_{\infty }$ denote the ignition temperature of the sample and ambient temperature (K), respectively.

In this work, the TTI for the five samples of P1A0, P3A1, P1A1, P1A3 and P0A1 were calculated to be 688, 687, 687, 692 and 701 K based on Equation 4 using a MATLAB program.

#### 3.2.2 Mass loss rate

**FIGURE 4:**
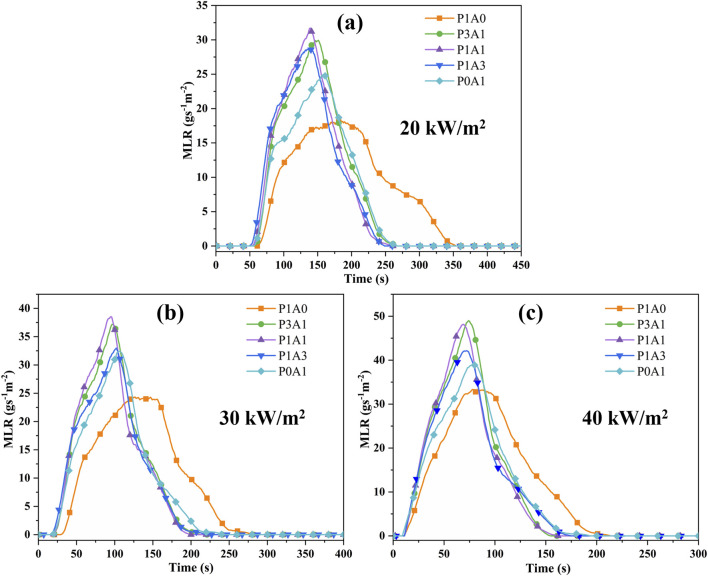
Plots of MLR vs. time for the five samples at: **(a)** 20 kW/m^2^, **(b)** 30 kW/m^2^ and **(c)** 40 kW/m^2^.

The MLR (Chen et al., 2019; Chen et al., 2014) is the rate of mass loss during the vaporization and combustion of a solid or liquid fuel. MLR can be used to profile the decomposition rate of a sample and assess its fire risk. Figure 4 demonstrates the MLR versus time for the five samples with radiation heat flux.

From Figure 4, the p-MLR and the a-MLR increase with increasing heat flux. According to the literature (Rhodes and Quintiere, 1996), the transient MLR can be expressed as Equation 5:

$$m^{\prime \prime }=\left(q_{e}^{\prime \prime }+q_{f,c}^{\prime \prime }+q_{f,r}^{\prime \prime }-q_{cond}^{\prime \prime }-\sigma \left(T_{V}^{4}-T_{\infty }^{4}\right)\right)/L$$ (5)

where $m^{\prime \prime }$ is the transient MLR of the sample (gs^−1^m^−2^). *L* is the latent heat of the vaporization of the sample (kJ/g). $q_{f,c}^{\prime \prime }$ and $q_{f,r}^{\prime \prime }$ are the convective and radiant heat fluxes of the flame (kW/m^2^), respectively. $q_{cond}^{\prime \prime }$ is the heat transfer loss of the sample (kW/m^2^). $\sigma \left(T_{V}^{4}-T_{\infty }^{4}\right)$ is the secondary radiative heat loss from the sample surface (kW/m^2^). *T*_*V*_ is the vaporization temperature of the sample (K).

**FIGURE 5:**
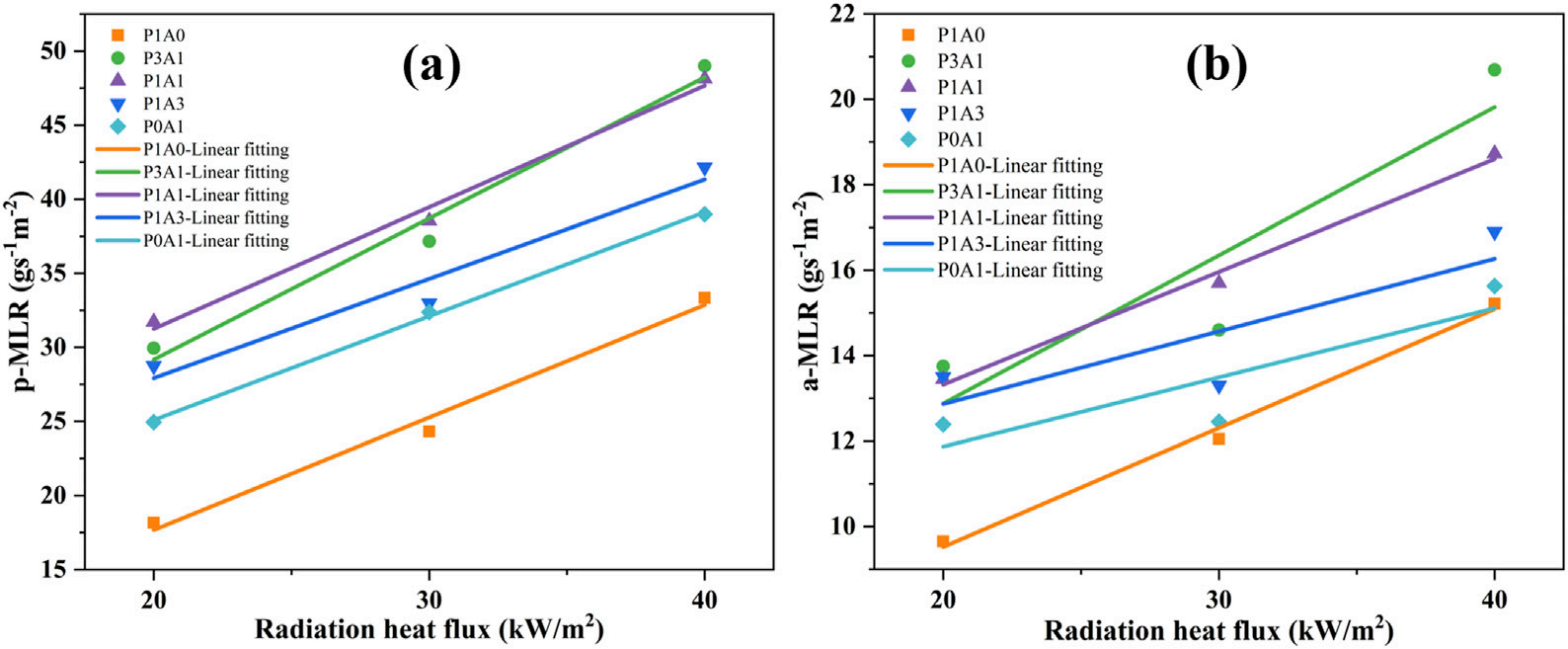
Plots of MLR versus heat flux for the five samples: **(a)** p-MLR and **(b)** a-MLR.

Both the $q_{f,c}^{\prime \prime }$ and $q_{f,r}^{\prime \prime }$ of the flame at steady-state (or quasi-steady-state) combustion or p-MLRs can be considered approximately constant for a given sample and sample size as per literature (Quintiere, 2006; Janssens et al., 2003; Rhodes and Quintiere, 1996; Hopkins and Quintiere, 1996; Quintiere and Rangwala, 2004). The $q_{cond}^{\prime \prime }$ of a sample can be explained as the heat of vaporization of the sample at steady state (or quasi-steady state) combustion or p-MLR. The *T*_*V*_ can be approximated as being equal to the ignition temperature of the sample. For a given sample and sample size, the TTI of the sample is constant. Therefore, all terms on the right-hand side of the equation at steady-state (or quasi-steady-state) combustion or p-MLR are constant except for *q*_*e*_ *''* as shown in Equation 6. The p-MLR and a-MLR can be linearly correlated with the *q*_*e*_ *”*.

$$m^{\prime \prime }=q_{e}^{\prime \prime }/L+C$$ (6)

where *C* is a constant. Figure 5 shows the p-MLR and a-MLR as a function of heat flux. Figure 5a shows a good linear fit of the p-MLR to the heat flux. The linear relationship between a-MLR and heat flux is not so well shown in Figure 5B. The correlation is expressed as a relationship as shown in Table 6. $m_{p}^{\prime \prime }$ and $m_{a}^{\prime \prime }$ denote the p-MLR and a-MLR respectively. *L*_*p*_ and *L*_*a*_ represent the latent heat of vaporization of the sample calculated from the p-MLR and a-MLR (kJ/g), respectively.

**TABLE 6:** Summary of MLR-related data for the five samples.

Polymer	Equation	R^2^	L_p_ (kJ/g)	Equation	R^2^	L_a_ (kJ/g)
P1A0	*m*_*p*_ *”* = 0.760*q*_*e*_ *”*+2.482	0.99	1.32	*m*_*a*_ *”* = 0.279*q*_*e*_ *”*+3.952	0.99	3.58
P3A1	*m*_*p*_ *”* = 0.953*q*_*e*_ *”*+10.110	0.98	1.05	*m*_*a*_ *”* = 0.347*q*_*e*_ *”*+5.937	0.84	2.88
P1A1	*m*_*p*_ *”* = 0.821*q*_*e*_ *”*+14.832	0.99	1.22	*m*_*a*_ *”* = 0.264*q*_*e*_ *”*+8.040	0.99	3.79
P1A3	*m*_*p*_ *”* = 0.670*q*_*e*_ *”*+14.519	0.96	1.49	*m*_*a*_ *”* = 0.170*q*_*e*_ *”*+9.467	0.71	5.88
P0A1	*m*_*p*_ *”* = 0.701*q*_*e*_ *”*+11.064	1.00	1.43	*m*_*a*_ *”* = 0.162*q*_*e*_ *”*+8.630	0.76	6.17

The latent heat of vaporization L of the specimen is the inverse of the slope of the fitted straight line according to Equation 6; Figures 4, 5; Table 6. The fitted lines of p-MLR with heat fluxes for P1A0, P3A1, P1A1, P1A3 and P0A1 calculated *L*_*p*_ of 1.32, 1.05, 1.22, 1.49 and 1.43 MJ/kg, respectively. The fitted lines of a-MLR with heat fluxes for P1A0, P3A1, P1A1, P1A3 and P0A1 calculated *L*_*a*_ of 3.58, 2.88, 3.79, 5.88 and 6.17 MJ/kg, respectively. The *L*_*a*_ calculated for the same sample a-MLR is much larger than the *L*_*p*_ calculated for the p-MLR. This may be caused by the fact that the whole thermal decomposition process of PLA, ABS and PLA/ABS cannot be considered as steady-state or quasi-steady-state combustion. The a-MLR cannot be considered as strictly following the linear relationship with a heat flux of Equation 7. These resulted in poor confidence in *L*_*a*_ calculated from the fitted lines of a-MLR and heat flux (Chen et al., 2015b). Therefore, the heat of vaporization *L*_*p*_ calculated from the fitted line of p-MLR and heat flux was used in this study for subsequent analysis.

#### 3.2.3 Heat release rate

**FIGURE 6:**
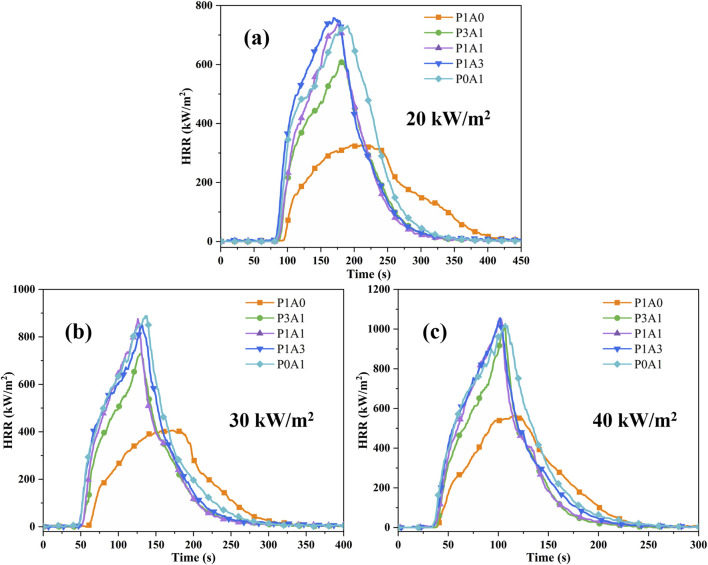
Plots of HRR for the five samples: **(a)** 20 kW/m^2^, **(b)** 30 kW/m^2^ and **(c)** 40 kW/m^2^.

The HRR of a sample characterizes the rate at which heat is released by the sample burning, which is considered to be the most important variable in fire risk assessment (Carpenter and Janssens, 2005). Figure 6 shows a plot of HRR versus time for the five samples at different heat fluxes.

As seen in Figure 6; Table 4, pure P1A0 starts to burn after ignition and reaches a plateau between 160 and 250 s, followed by a gradual decrease in HRR values to 0 under radiation heat flux conditions of 20 kW/m^2^. P3A1, P1A1, P1A3 and P0A1 burn faster on ignition. P3A1 reaches a PHRR of 608 kW/m^2^ at 182 s. P1A1, P1A3 and P0A1 have similar curve shapes and similar PHRR values. The PHRRs for these three samples are 738, 758 and 732 kW/m^2^ respectively. This means that the percentage of ABS determines the PHRR of PLA/ABS. The PLA/ABS blend gives a similar HRR to that of pure ABS when the PLA content is 50%.

**FIGURE 7:**
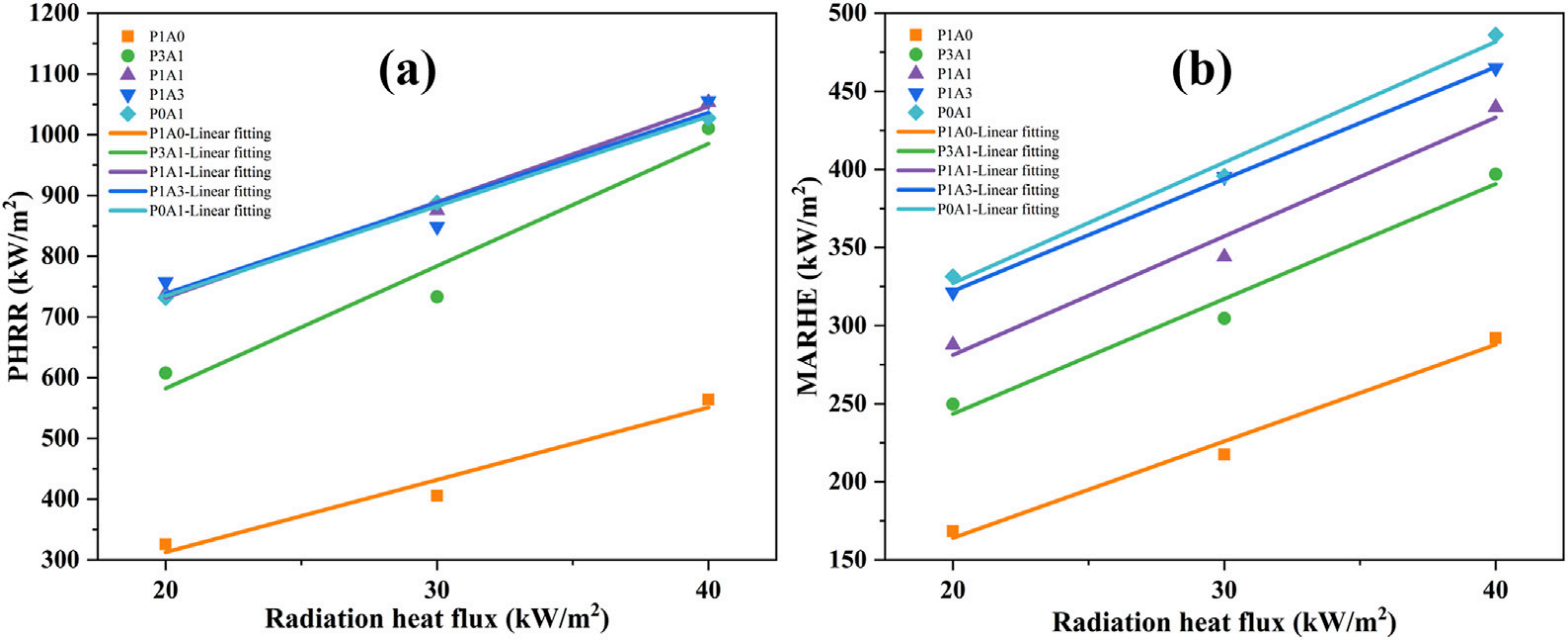
Plots of HRR parameters versus heat flux for the five samples: **(A)** PHRR, **(B)** MARHE.

As shown in Figure 7, the PHRR and MARHE increased with increasing heat flux for the five samples. The transient HRR can also be obtained from the transient MLR and the theoretical heat of combustion of the sample in addition to the heat of oxygen consumption method (Quintiere, 2006):

$${q^{\prime \prime }=m}^{\prime \prime }{∆H}_{c}^{T}={∆H}_{c}^{T}\left(q_{e}^{\prime \prime }+q_{f,c}^{\prime \prime }+q_{f,r}^{\prime \prime }-q_{cond}^{\prime \prime }-\sigma \left(T_{V}^{4}-T_{\infty }^{4}\right)\right)/L$$ (7)

where $q^{\prime \prime }$ is the transient HRR (kW/m^2^). ${∆H}_{c}^{T}$ is the theoretical heat of combustion of the sample (kJ/g), which is the standard heat given off per unit mass of the sample for complete combustion. As per the literature, the standard heat of combustion determined using an oxygen bomb calorimeter for PLA was measured to be 19 MJ/kg (Knurr and Hauri, 2020) and for ABS to be 39 MJ/kg (Walters et al., 2000).

The p-MLR and a-MLR are linearly related to the heat flux, as shown in section 3.2.2Mass Loss Rate. The HRR is proportional to MLR for a given sample when the ${∆H}_{c}^{T}$ remains constant according to Equation 7. Thus, both the PHRR and the MARHE during the quasi-steady-state phase can be linearly related to the heat flux as p-MLR and a-MLR, as shown in Equation 8.

$$q^{\prime \prime }=m^{\prime \prime }{∆H}_{c}^{T}=q_{e}^{\prime \prime }{∆H}_{c}^{T}/L+C_{1}$$ (8)

where *C*_*1*_ is a constant. As shown in Equation 8, the ${∆H}_{c}^{T}$ can be obtained from the slope of a linear fit of PHRR and MARHE to the change in heat flux during steady state (or quasi-steady state) combustion of the HRR. As shown in Figure 7, PHRR and MARHE are functions of heat flux. The equations for PHRR and MARHE versus heat flux are listed in Table 7. The *q*_*p*_ *''* and *q*_*m*_ *''* are PHRR and MARHE, respectively.

**TABLE 7:** Summary of heat release rate parameters for the five samples.

Polymer	Equation	R^2^	ΔH_c_^T^	Equation	R^2^	ΔH_c_^T^ (MJ/kg)
P1A0	*q*_*p*_ *”* = 11.917*q*_*e*_ *”* + 74.307	0.96	15.73	*q*_*m*_ *”* = 6.19*q*_*e*_ *”* + 40.233	0.99	8.17
P3A1	*q*_*p*_ *”* = 20.146*q*_*e*_ *”* + 179.373	0.95	21.15	*q*_*m*_ *”* = 7.365*q*_*e*_ *”* + 96.15	0.98	7.73
P1A1	*q*_*p*_ *”* = 15.737*q*_*e*_ *”* + 417.047	0.99	19.20	*q*_*m*_ *”* = 7.605*q*_*e*_ *”* + 129.05	0.98	9.28
P1A3	*q*_*p*_ *”* = 14.889*q*_*e*_ *”* + 440.768	0.95	22.18	*q*_*m*_ *”* = 7.175*q*_*e*_ *”* + 178.617	0.99	10.69
P0A1	*q*_*p*_ *”* = 14.781*q*_*e*_ *”* + 438.953	1	21.14	*q*_*m*_ *”* = 7.73*q*_*e*_ *”* + 172.567	0.99	11.05

Based on Equation 8; Table 7, the ${∆H}_{c}^{T}$ for the five samples was calculated by combining the theoretical values of the heat of vaporization (*L*_*p*_) calculated from the MLR data in Section 3.2.2 Mass Loss Rate and are shown in Table 7. The ${∆H}_{c}^{T}$ obtained by fitting PHRR to radiation heat fluxes for P1A0, P3A1, P1A1, P1A3 and P0A1 were 15.73, 21.15, 19.20, 22.18 and 21.14 MJ/kg respectively. The ${∆H}_{c}^{T}$ obtained by fitting P1A0, P3A1, P1A1, P1A3 and P0A1 to the radiation heat flux by MARHE were 8.17, 7.73, 9.28, 10.69 and 11.05 MJ/kg, respectively. As reported earlier in section 3.2.2 Mass Loss Rate section and Figure 6, the entire decomposition process of the sample cannot be considered a steady-state or quasi-steady-state phase. Therefore, it may be more reasonable to choose PHRR data rather than MARHE data to calculate the ${∆H}_{c}^{T}$ The ${∆H}_{c}^{T}$ found by fitting PHRR to radiation heat fluxes are closer to the heat of combustion measured using an oxygen bomb calorimeter (19 MJ/kg for PLA). Incomplete combustion of samples and uncertainties in measurement and calculation methods can cause differences in values. The more reliable theoretical heat of combustion is obtained from the theoretical heat of vaporization (L_p_) calculated from the peak MLR data. This can indicate that the accuracy of the theoretical heat of vaporization calculated from the peak MLR data is acceptable.

If there is no interaction between the components of the blend, a combination of Equation 1; Equation 8 is obtained from the ratio of the heat released from the combustion of the sample alone to the mass of the components in the blend. This equation is expressed as

$$q^{\prime \prime }/{\Delta H}_{c}^{T}=X_{1}q_{1}^{\prime \prime }/{\Delta H}_{c1}^{T}+X_{2}q_{2}^{\prime \prime }/{\Delta H}_{c2}^{T}$$ (9)

**FIGURE 8:**
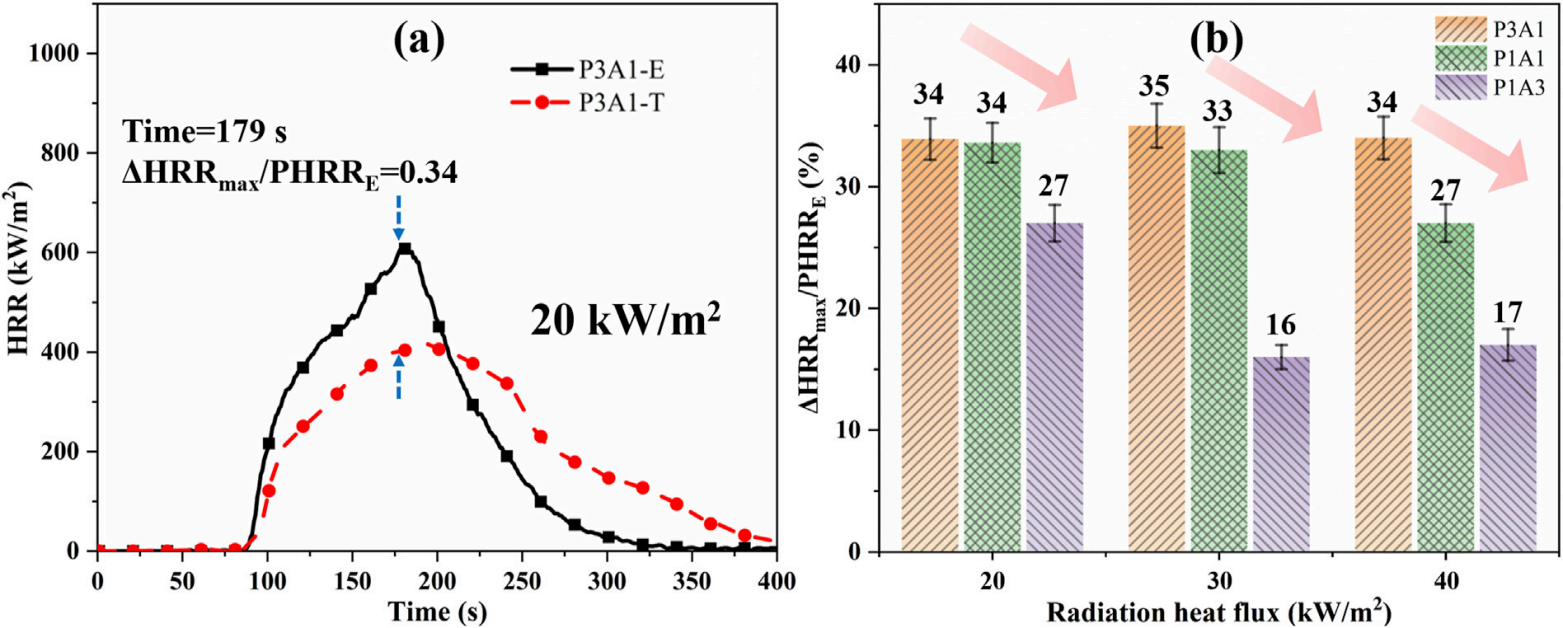
A comparison of theoretical (T) and experimental (E) HRR for PLA/ABS blends: **(a)** the HRR curve for P3A1 at 20 kW/m^2^ and **(b)** ΔHRR_max_/PHRR_E_.

The theoretical HRRs for the five samples were calculated from Equation 9, as shown by the dashed lines in Figure 8a. Figure 8b shows the value of ΔHRR_max_/PHRR_E_ for different samples at three radiation heat fluxes. As shown in Figure 8a, the theoretical and experimental HRR curves do not exactly overlap, while the experimental sample has a faster rise in HRR and a higher PHRR. This indicates that PLA and ABS are more than just the sum of the two burnings separately in the combustion process. From Figure 8b, the ratio ΔHRR_max_/PHRR_E_ of P3A1, P1A1 and P1A3 are in the ranges of 34%–35%, 27%–34% and 16%–27% at different radiant heat fluxes. The sample P3A1 has the largest difference in the cone calorimetry. The presence of a small amount of ABS in the blend causes a more pronounced enhancement of the pyrolysis and combustion processes, leading to a higher HRR compared to theoretical results. As the percentage of ABS in the blend increases, the difference between experiment and theory decreases. This is the opposite of the enhanced pyrolysis-promoting effect of PLA/ABS as ABS increases as per Section 3.1 Co-pyrolysis behaviors. This indicates that the degree of burning of the mixture cannot be deduced from the degree of a pyrolytic reaction of the mixture alone. The reason for this opposite trend may be because the more PLA there is in the TGA tests, the lower the temperature corresponding to the second peak reflecting ABS pyrolysis relative to the theoretical value. PLA catalyzes the pyrolysis of ABS in the lower temperature region, resulting in a more vigorous combustion reaction. These results reveal the significant influence of component interactions on the combustion behavior of PLA/ABS blends, providing new insights into their thermal degradation mechanisms.

#### 3.2.4 Total heat release and effective heat of combustion

THR is the total heat energy released when a solid combustible sample burns. EHC represents the actual heat released per unit mass of the sample burned (Santoni et al., 2015). THR and EHC can be calculated using Equations 10, 11 below.

$$\text{THR}=\int_{t=0}^{t_{end}}q^{\prime \prime }$$ (10)

**FIGURE 9:**
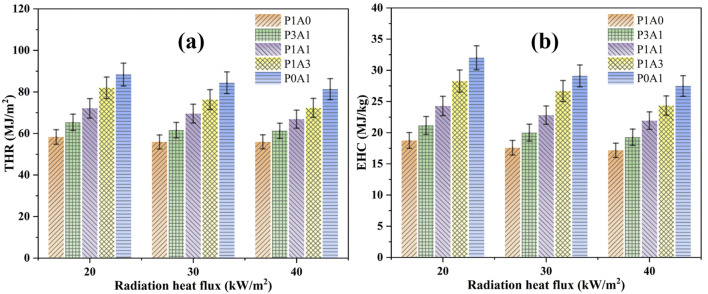
Histograms of five samples at three radiation heat fluxes: **(a)** THR and **(b)** EHC.

EHC=THR/Δm (11)

where *t*_*end*_ to denote the end time of combustion (s). The *Δm* is the sample mass loss at the ignition end of combustion (g). Figure 9 shows the THR and EHC histograms for the five samples at three radiation heat fluxes. The results show that the THR values increase uniformly with the proportion of ABS components in the mixture. The THR of the same sample remains almost constant with increasing radiation heat flux. This indicates that THR is an intrinsic property of the sample. The THR of a mixture corresponds to the percentage of each substance. Similar to THR, EHC increases uniformly as the percentage of ABS components in the mixture increases. The EHC value for the same sample remains almost constant with increasing radiation heat flux. The small difference between the values may be due to incomplete combustion, which is strongly influenced by the amount of oxygen in the combustion zone.

The average EHC for P1A0, P3A1, P1A1, P1A3 and P0A1 at the three different heat fluxes were 18, 20, 23, 26 and 29 MJ/kg respectively according to Figure 9; Table 4. This is close to the theoretical EHC as reported earlier in Section 3.2.3 Heat release rates of 15.73, 21.15, 19.20, 22.18 and 21.14 MJ/kg for P1A0, P3A1, P1A1, P1A3 and P0A1 respectively. The average EHC of PLA (18 MJ/kg) is almost identical to the theoretical heat of combustion (19 MJ/kg) obtained with an oxygen bomb calorimeter. The average EHC of the ABS (29 MJ/kg) differs somewhat from the theoretical heat of combustion (39 MJ/kg) obtained with an oxygen bomb calorimeter. This difference can be attributed to incomplete combustion due to insufficient oxygen during combustion. The average EHC for five samples at the three different heat fluxes is much greater than the theoretical heat of combustion deduced from the MARHE data of 8.17, 7.73, 9.28, 10.69 and 11.05 MJ/kg for P1A0, P3A1, P1A1, P1A3 and P0A1, respectively. This could further indicate that the entire thermal decomposition of the sample in this study cannot be considered a steady-state phase. Based on this study, the average HRR data may not be used to estimate the theoretical heat of combustion. Instead, the peak HRR data can be used to obtain relatively accurate theoretical heats of combustion for the thermal decomposition of the five samples.

## 4 Conclusion

This study investigates the co-pyrolysis and combustion properties of PLA/ABS blends using TGA and cone calorimetry. The results indicate that PLA catalyzes the pyrolysis of ABS in blends, leading to a more vigorous combustion reaction. The presence of ABS in the blend enhances the thermal degradation of PLA, resulting in a significant synergistic effect on HRR and THR. The kinetic analysis shows that the interaction between PLA and ABS accelerates the degradation process, particularly at lower temperatures. These findings have implications for the efficient utilization and fire protection of PLA/ABS blends. Future work will focus on further exploring the mechanisms of these interactions and improving the mechanical properties of PLA/ABS blends.

## Data Availability

The datasets presented in this article are not readily available because the dataset has been provided in the manuscript. Requests to access the datasets should be directed to fei.xiao@whut.edu.cn.
